# Unravelling Cotton Nonexpressor of Pathogenesis-Related 1(NPR1)-Like Genes Family: Evolutionary Analysis and Putative Role in Fiber Development and Defense Pathway

**DOI:** 10.3390/plants9080999

**Published:** 2020-08-06

**Authors:** Neha Agarwal, Rakesh Srivastava, Akash Verma, Krishan Mohan Rai, Babita Singh, Praveen Chandra Verma

**Affiliations:** 1 Molecular Biology and Biotechnology Division, CSIR-National Botanical Research Institute, Council of Scientific and Industrial Research, Lucknow 226001, India; agarwalneha290@gmail.com (N.A.); gsvakash@gmail.com (A.V.); babita.singh009@gmail.com (B.S.); 2 Academy of Scientific and Innovative Research (AcSIR), Ghaziabad 201002, India; 3 Department of Plant and Microbial Biology, University of Minnesota, St. Paul, MN 55108, USA; Kmohanra@umn.edu

**Keywords:** NPR1, gene expression, fiber development, plant defense, cis-regulatory elements

## Abstract

The nonexpressor of pathogenesis-related 1 (NPR1) family plays diverse roles in gene regulation in the defense and development signaling pathways in plants. Less evidence is available regarding the significance of the *NPR1*-like gene family in cotton (*Gossypium* species). Therefore, to address the importance of the cotton *NPR1*-like gene family in the defense pathway, four *Gossypium* species were studied: two tetraploid species, *G.* *hirsutum* and *G. barbadense*, and their two potential ancestral diploids, *G. raimondii* and *G. arboreum.* In this study, 12 *NPR1*-like family genes in *G. hirsutum* were recognized, including six genes in the A-subgenome and six genes in the D-subgenome. Based on the phylogenetic analysis, gene and protein structural features, cotton NPR-like proteins were grouped into three different clades. Our analysis suggests the significance of cis-regulatory elements in the upstream region of cotton *NPR1*-like genes in hormonal signaling, biotic stress conditions, and developmental processes. The quantitative expression analysis for different developmental tissues and fiber stages (0 to 25 days post-anthesis), as well as salicylic acid induction, confirmed the distinct function of different cotton *NPR* genes in defense and fiber development. Altogether, this study presents specifications of conservation in the cotton *NPR1*-like gene family and their functional divergence for development of fiber and defense properties.

## 1. Introduction

The sessile nature of plants makes them susceptible to sporadicity in environmental and biotic stresses. To protect themselves against intruding pathogens, plants have evolved several barrier systems for defense. Systemic acquired resistance (SAR) is an induced immune mechanism activated against a broad spectrum of pathogens and provides long-lasting immunity to the distal uninfected tissues from the synchronized activation of various pathogenesis-related genes [1,2]. Salicylic acid (SA) is a crucial signaling molecule for the initiation of the SAR response and expression of pathogenesis-related genes (PRs) [3,4]. Screening of *Arabidopsis* mutants that are dysfunctional in mounting salicylic acid-mediated defense response leads to the identification of a master regulator of the SAR pathway, NPR1 (nonexpressor of pathogenesis-related 1) [5]. Interestingly, NPR1 has not only emerged as the major active player for the plant defense response but also functions downstream of SA signaling to regulate salicylic acid biosynthesis [6,7,8]. In the nucleus, NPR1 functions as a transcription co-activator, together with TGA transcription factors, to facilitate the transcriptional activation of the SAR genes [9]. In uninduced conditions, a small quantity of NPR1 is constitutively localized in the nucleus and is subjected to proteasomal degradation to avoid unnecessary defense gene activation, whereas pathogenic infection or salicylic acid induction promotes NPR1 monomerization and nuclear translocation. In induced conditions, NPR1 degradation promotes its rapid turnover and augments gene expression in the SAR pathway [10].

Hepworth et al., 2005, classified six homologs of the *NPR1*-like gene family (*AtNPR1*, *AtNPR2*, *AtNPR3*, *AtNPR4*, *AtNPR5*, and *AtNPR6*) in three different clades based on the phylogenetic analysis in *Arabidopsis thaliana* [11]. Many reports argue that several plant species ectopically expressing *AtNPR1* display improvised resistance against a broad spectrum of bacterial and fungal pathogens, for instance, wheat [12], citrus [13,14], rice [15,16], tomato [17], rapeseed [18], tobacco [19], carrot [20], and cotton [21,22,23], due to enhanced activation of defense pathway genes. In recent years, whole-genome sequencing of many plants has led to the characterization of homologs in avocado [24], papaya [25], wheat [26], apple [27], and strawberry [28].

Cotton is an economically valuable industrial crop. All over the world, it is mostly grown for oil-rich seeds and fiber, which is a required raw material for the textile industry. Biotic stress is an important constraint that inhibits cotton growth and development and compromises its fiber quality and yield [29]. Application of insecticidal spray or a transgenic variety leads to the development of field-evolved pathogen resistance, which is a sustainable option. An alternative application could be exploration of inherent or induced disease-resistant mechanisms in cotton, for example, demystifying the *NPR1*-like gene family of the SAR pathway, which would be an essential advantage for providing resistance against pathogen infestation [30]. However, genomic information and functional profiles of the cotton *NPR* gene family is still unexplored. Recently, published draft genome sequences of *Gossypium hirsutum, Gossypium barbadense, Gossypium arboreum,* and *Gossypium ramondii* have opened up new avenues to explore genome-wide analysis of NPR family members after SA induction or developmental signaling in cotton [31,32,33,34]. In the present work, characterization of the *NPR1*-like gene family in four *Gossypium* species—*G. arboreum, G. barbadense, G. hirsutum,* and *G. ramondii*—was done. Further, gene structure, protein domain, phylogenetic analysis, and synteny of the identified putative cotton *NPR* genes were also analyzed. This study will provide an essential source for utilizing the *NPR1*-like genes in cotton for developing inherent resistance or tolerance against pathogens, and will eventually help us in developing higher fiber yielding varieties, an important asset for the cotton industry.

## 2. Results

### 2.1. Identification and Classification of NPR Homologs in Cotton

**Figure 1:**
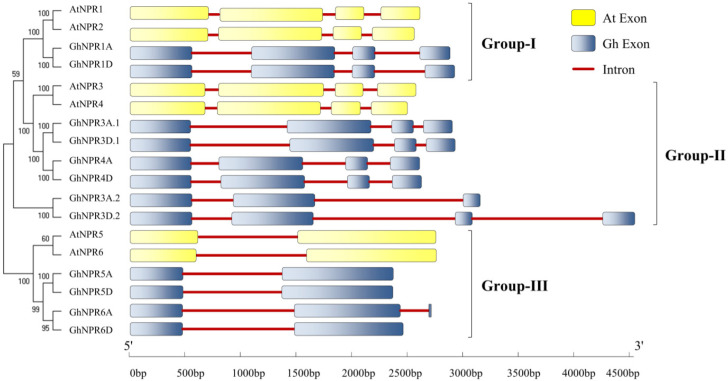
Classification of the *Gossypium hirsutum NPR1-*like gene family. Phylogenetic relationships were inferred between 12 *GhNPR* genes and 6 *AtNPR* genes through the neighbor-joining method. The exon-intron structure for *G. hirsutum* and *Arabidopsis* are represented in blue and yellow boxes, respectively, whereas their introns are represented by red lines.

**Figure 2:**
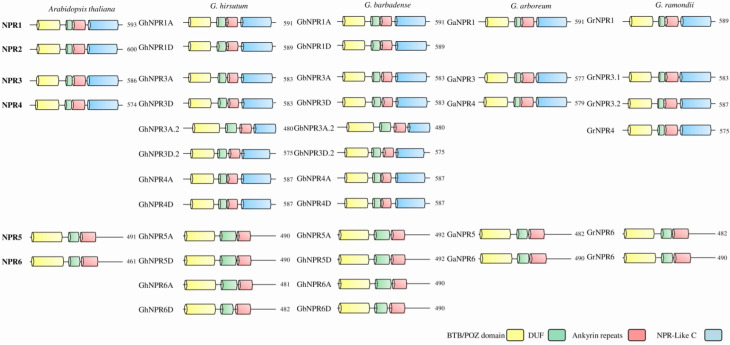
Schematic representation of domain organization of NPR1-like proteins in *Arabidopsis* and *Gossypium* species. The positions of conserved domains like BTB/POZ (broad complex, tramtrack, and bric a brac/pox virus and zinc finger), ankyrin repeats, DUF (domain of unknown function), and NPR1-like C-terminal region (transactivation domain) were shown for *A. thaliana*, *G. hirsutum*, *G. barbadense*, *G. arboreum,* and *G. ramondii*. Number represents amino acid of protein derived from NCBI accession numbers (Table S2).

Six *Arabidopsis* NPR (AtNPR1-6) protein sequences were used as queries in the BLASTP protein database for genomes of two allotetraploid cotton species, *G. hirsutum,* and *G. barbadense*, and their two diploid ancestors, *G. arboreum* and *G. ramondii* (Table 1, Table S2). Twelve putative *NPR1*-like genes were identified in both allotetraploids *G. hirsutum* and *G. barbadense*, whereas five and six *NPR1*-like genes were identified in diploids *G. arboreum* and *G. ramondii,* respectively (Figure 1 and Figure 2, Figure S1 and Table 1). The *Arabidopsis* and cotton species NPR homologs share high protein similarity, which ranges from 75% to 91% (Table S3). To annotate 35 putative *NPR* genes, the nomenclature was based on high protein sequence similarity and domain similarity (Figure 2 and Table 1). The identified cotton species *NPR* genes were further confirmed by the existence of two highly conserved protein domains, BTB/POZ and ankyrin repeat (ANK) domains, which are indicative domains present in the members of *Arabidopsis* NPR and other plants’ NPR families (Figure 2) [24,25,26,27,28]. All the domains of the *Arabidopsis* NPR1 family, BTB/POZ, DUF, ANK, and the C-terminal domain NPR1_like_C (transactivation domain) are present in the NPR1-like proteins of cotton species. The domains of the cotton NPR components share high similarity with their *Arabidopsis* counterparts.

### 2.2. Phylogenetic and Synteny Analysis of Cotton NPR1-Like Protein Family

**Figure 3:**
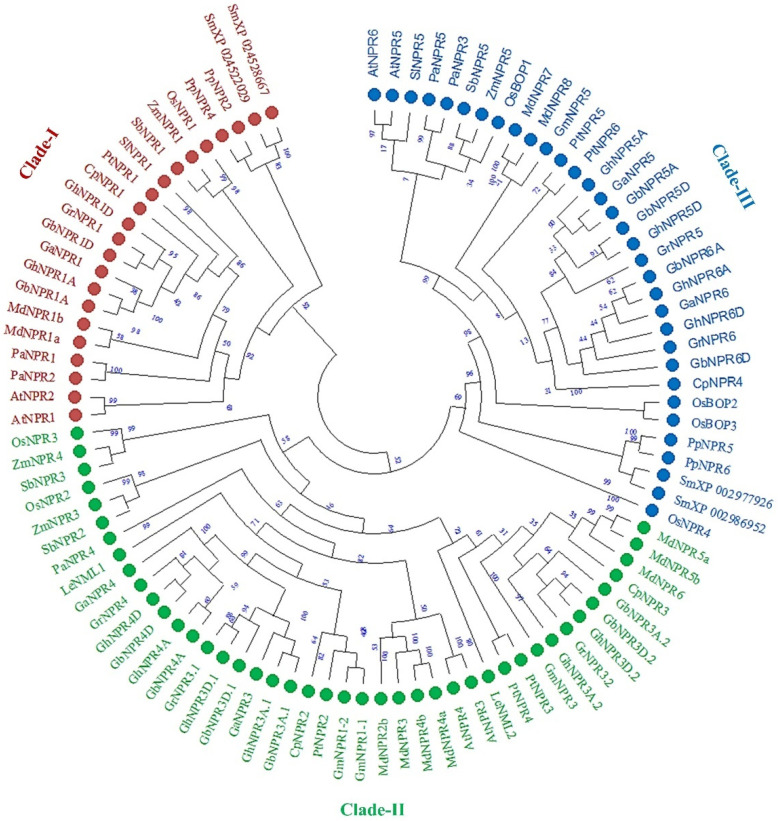
Phylogenetic and evolutionary relationships of *Gossypium* NPR family. Phylogenetic tree was constructed by MEGA-X with 1000 bootstrap replicates by using full-length protein sequences from At, *Arabidopsis thaliana*; Pa, *Persea americana*; Ca, *Carica papaya*; Gm*, Glycine max*; Md, *Malus domestica*; Pt, *Populus trichocarp*a; Le, *Lycopersicon esculentum*; Sb, *Sorghum bicolor*; Os, *Oryza sativa*; Zm, *Zea mays*; Pp *Physcomitrella patens*; and Sm, *Selaginella moellendorffii*. Number on branches is indicative of the bootstrap values. Red circle, green circle, and blue circle represent clade I, clade II, and clade III, respectively.

In order to gain insight into evolutionary association and phylogenetic relationships of the NPR1-like protein family in cotton, a phylogenetic tree was generated using full-length protein sequences of dicot plants (*A. thaliana*, *Persea americana*, *Carica papaya*, *Glycine max*, *Malus domestica*, *Populus trichocarpa,* and *Lycopersicon esculentum*), monocot plants (*Sorghum bicolor*, *Oryza sativa,* and *Zea mays*), bryophytes (*Physcomitrella patens*), and lycophytes (*Selaginella moellendorffii*) (Table S2). The phylogenetic analysis showed that all putative *Gossypium* NPR1-like proteins were divided into three clades based on the homologous sequence. In clade I, all the NPR1 homologs in *Gossypium* species were located in a similar cluster with AtNPR1 and AtNPR2. Similarly, in *Gossypium* species NPR3 and NPR4 marked their presence with AtNPR*3* and AtNPR4 in clade II. In clade III, *Gossypium* species NPR5 and NPR6 were clustered together with AtNPR5 and AtNPR6 (Figure 3). The approach of elucidating three groups of cotton NPR1-like protein was also similar to previous reports in *Arabidopsis*, papaya, apple, or avocado [11,25,27,35]. In all the three groups, members belonging to dicots or monocots were clustered together, while bryophytes and pteridophytes forming a similar cluster. The NPR1-like proteins homologous to *Gossypium* species were closely clustered together with different dicots than with the members of monocot species, indicating closer relationships with members of dicots as compared to monocots. Further, NPR1-like proteins from *Gossypium* species were closer to tree-like plants such as *C. papaya*, *M. domestica*, and *P. trichocarpa* (Figure 3).

**Figure 4:**
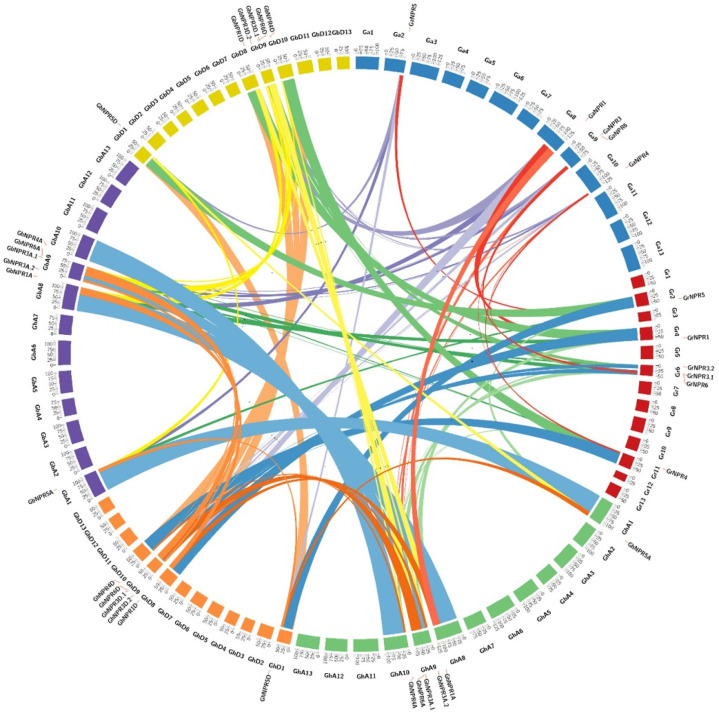
Syntenic relationship of *Gossypium NPR* genes. Synteny analysis between *G. hirsutum*, *G. barbadense*, *G. arboreum,* and *G. ramondii NPR* gene family.

The *NPR1*-like genes are mostly located on four chromosomes of the *G. hirsutum* A- and D-subgenomes (Figure S2). Events of gene duplication have an effect on the amplification of gene families. The allotetraploid cotton species *G. hirsutum, and G. barbendense* originated from *G. raimondii* and *G. arboreum*. The event of gene duplication was executed across the four genomes of *G. hirsutum, G. barbendense, G. arboreum,* and *G. raimondii* (Figure 4). The duplicate genes of the *NPR1*-like gene family in the two allotetraploid cotton species were categorized into segmental duplication (Figure 4).

### 2.3. Cotton NPR1-Like Gene Family Protein and Gene Structural Properties

**Figure 5:**
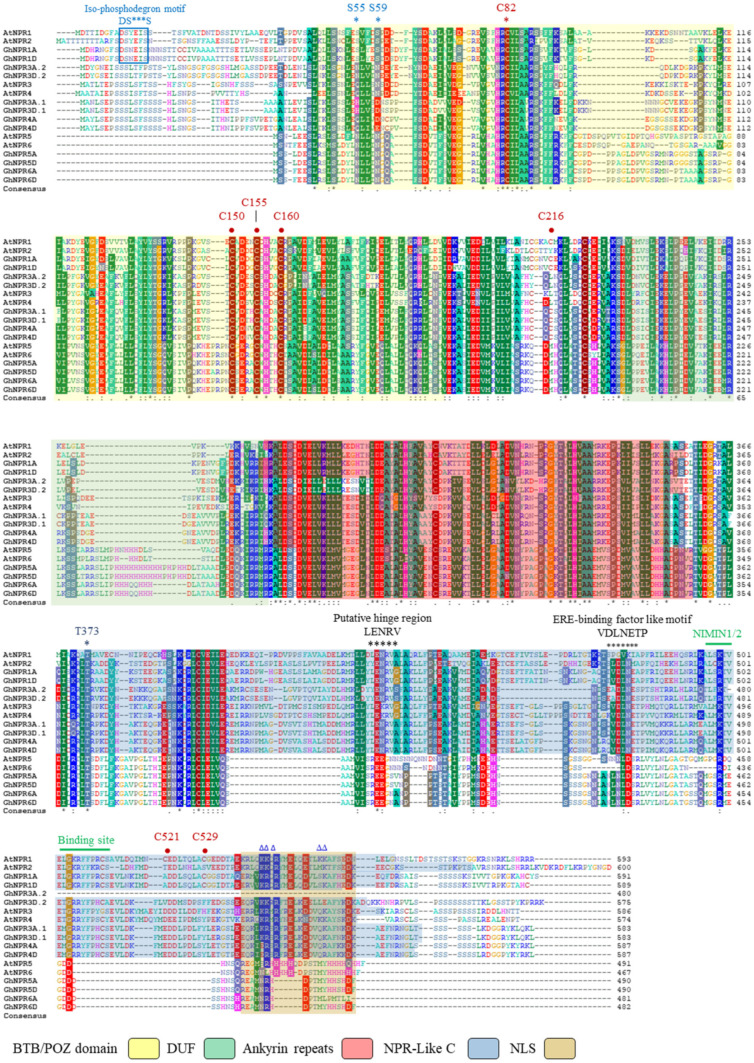
Multiple sequence alignment of GhNPR proteins with *Arabidopsis*. The BTB/POZ (yellow background), DUF (green background), ankyrin repeat (red background), NPR1-like C-terminal region (blue background) and nuclear localization signal (NLS; brown background) for each individual GhNPR and AtNPR. Important motifs, like IκB phosphodegron, putative hinge region (LENRV), ERE binding factor-like motif (VDLNTEP), and NIMIN1/2 binding site, are written over the motifs. Highly conserved cysteine residues (C82, C150, C155, and C160), serine residues (S55 and S59), threonine residues (T373) and basic amino acid (marked with Δ) of NLS1 effect on nuclear localization.

The results of domain architecture represent the fact that all the members of the cotton NPR family have BTB/POZ and ankryin repeat domains, almost at a similar position with reference to the *Arabidopsis* NPR family (Figure 5 and Figures S3–S5). The NPR1-like C-terminal region (a transactivation domain) is absent from cotton NPR5 and NPR6, similar to *Arabidopsis* AtNPR5 and AtNPR6 homologs, respectively (Figure 5 and Figures S3–S5). Similar to AtNPR1 and AtNPR2, certain essential serine phosphorylated sites, like S11, S15, S55, and S59, were also present in all putative cotton NPR1 homologs. All the cotton NPR homologs contain conserved cysteine residues at C82, C150, C155, and C160, yet only cotton NPR1A/1D possess conserved cysteine at 216 positions. These cysteine residues play an important role in maintaining the oligomeric state of NPR1 in the cytoplasm [36]. Furthermore, none of the cotton NPR proteins possess conserved cysteine residue at C521 and C529, which is required for the binding of salicylic acid through transition metal copper [7]. C521 is absent in GhNPR1A/D, whereas C529 is present in GhNPR1A/1D. All cotton NPR proteins, similar to *Arabidopsis*, have a conserved motif-like penta-amino acid (LENRV), VDLNETP motif, and NIMIN1/2 binding site at the C-terminus. Cotton NPR1, 3, and 4 contain three out of five conserved basic amino acid motifs in their nuclear localization signal (NLS) except for GhNPR4A.1, GhNPR4D.1, GbNPR4A.1, GbNPR4D.1, GrNPR4.1, and GaNPR4, which have four substitutions in this region (Figure 5 and Figures S3–S5) [37].

*GhNPR1*-like genes share a similar level of exon-intron structural organization of each gene, except for *GhNPR3A.2*, *GhNPR3D.2,* and *GhNPR6A*. Based on the exon-intron structure, the *NPR* genes were classified into three groups (Figure 1 and Figure S1). The homologs of *NPR* genes in groups I and II had four exons, whereas those in group III had two. Furthermore, the sizes of corresponding exons in each group were nearly similar. The protein charge of putative GhNPR proteins displays high variation and ranges from −8.5 to 4.0, suggesting the presence of acidic and basic amino acids (Table S4). However, theoretical pI varied from 5.3 to 6.7, indicating that these proteins could possess neutral side-chain amino acids. Consistently, protein charge and pI were observed in *G. barbadense, G. arboreum,* and *G. ramondii*. However, *Arabidopsis* showed a different nature of protein charge and pI in NPR family homologs. Furthermore, the presence of a negative grand average of hydropathy (GRAVY) score for cotton *NPR1*-like genes reflected their hydrophilic nature, revealing higher variability in hydrophilicity (Table S4).

### 2.4. Prediction of the Cis-Regulatory Elements in Cotton NPR1-Like Gene Family Promoter

**Figure 6:**
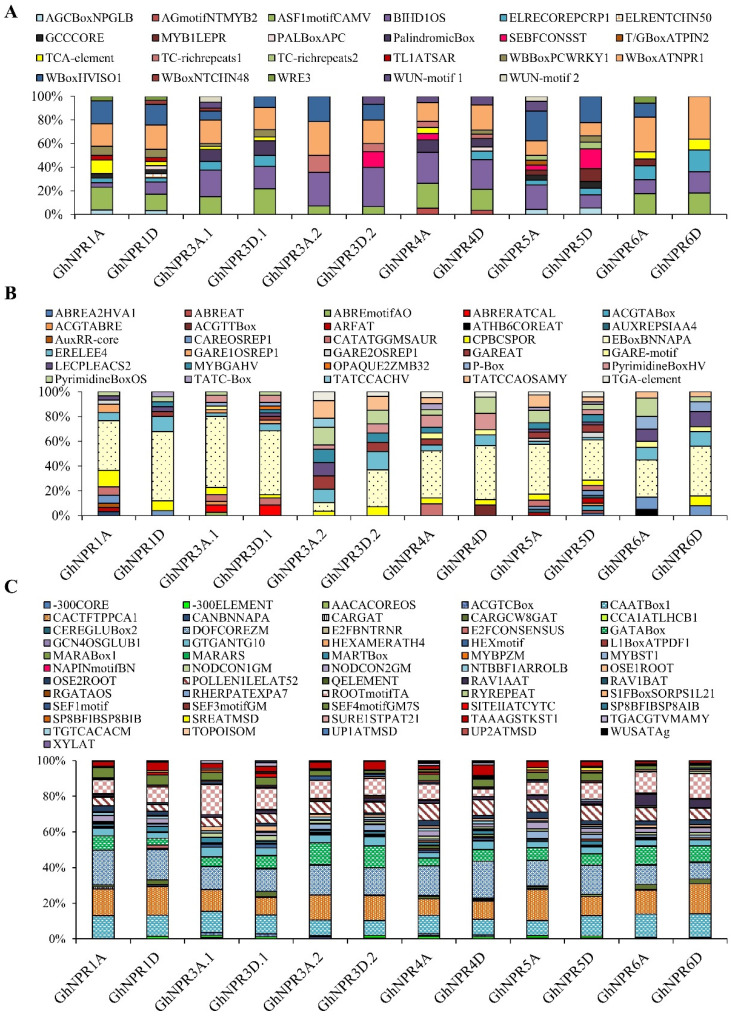
Analysis of the cis-regulatory elements in *GhNPR1*-like genes. Percentage of various cis-regulatory elements related to defense (**A**), hormone signaling (**B**), and development (**C**) present in the 2000 bp upstream region from the ATG site in *GhNPR* genes, identified through PLACE and PlantCare databases.

The occurrence of cis-regulatory element in the promoter of genes plays major roles in regulating gene expression during developmental or environmental changes. To examine the putative function of cotton *NPR1*-like genes, cis-regulatory elements in the 2000 bp upstream region from ATG (translational start codon) in the promoter of the *GhNPR* homologs in defense hormonal or stress conditions or developmental stages of cotton were analyzed from the PLACE and PlantCare databases (Figure 6, Table S5). The cis-regulatory element analysis suggested a large number of defense elements are present in *GhNPR1A/D*, *3A.1/D.1*, *4D*, and *5A* as compared with *GhNPR3A.2/D.2*, *4A*, *5D*, and *6A/D*. ASF1MOTIF, WRKY motif, and W-Box, well-known defense cis-regulatory elements, are generously present in *GhNPR1*, *3*, and *4* homologs compared to *GhNPR5/6* homologs (Figure 6A). Furthermore, hormone-related cis-regulatory elements were also profusely present in the upstream region of *GhNPR1*, *GhNPR3,* and *GhNPR5*, with comparatively fewer found in *GhNPR4* and *GhNPR6* homologs (Figure 6B). Additionally, the cis-regulatory elements associated with development were less present in *GhNPR1A*, *GhNPR1D*, *GhNPR3A.2,* and *GhNPR5D* as compared to others (Figure 6C). Furthermore, hormone-related cis-regulatory elements were also abundantly present in *GhNPR* genes, except for the *GhNPR4* and *GhNPR6* homologs. These results indicated that *GhNPR* homologs have important roles in the defense and hormonal signaling pathways, as well as developmental conditions, according to the prediction of in silico analysis on upstream regions of the cotton *GhNPR* genes.

### 2.5. In Silico Gene Expression Pattern of Cotton NPR1-Like Gene Family

**Figure 7:**
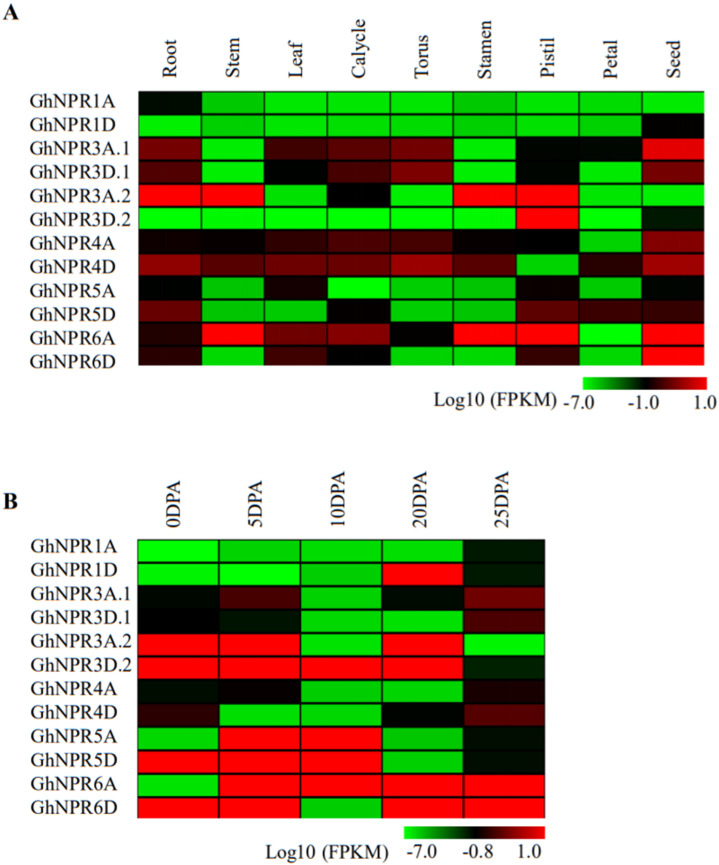
In silico expression analysis of *GhNPR1*-like genes of *G. hirsutum* in different plant tissues and fiber development stages. Heatmap represents the relative expression levels of 12 representative *GhNPR1*-like genes in various tissue (**A**) and fiber development stages (**B**). The scale bars at the bottom represent log10-transformed fragments per kilobase million (FPKM) values of each gene.

Understanding the gene expression pattern of identified *GhNPR* genes in different tissues can provide insightful information regarding their probable functional roles. To address gene expression patterns, we examined the expression profiles of *GhNPR* genes in different developmental stages, such as the leaf, calycle, stem, torus, stamen, root, pistil, petal, and seed stages using available transcriptome data [34] (Figure 7A). Most of the putative cotton *NPR1*-like genes showed either a very low or moderate level of transcripts. Interestingly, the lowest expression for *GhNPR1A/1D* genes was observed in all of the analyzed cotton tissues, suggesting no role in the development process. A higher expression was observed for *GhNPR3A.1* and *GhNPR6A/6D* in the seed stage, whereas *GhNPR3A.2/3D.2* and *GhNPR6A* were amongst the most highly expressed in the pistil stage. *GhNPR3A.2* was among the more expressed genes in the root. In tissues like the stem and stamen, there was a high expression of *GhNPR3A.2* and *GhNPR5A.2* genes.

We further analyzed the expression profiles for putative cotton *NPR1*-like genes at different cotton fiber development stages, such as 0 days post-anthesis (DPA), 5 DPA, 10 DPA, 20 DPA, and 25 DPA, from the available transcriptome data [34] (Figure 7B). Similarly, like in the cotton development tissue, we observed the lowest expression for *GhNPR1A/1D* genes in various fiber stages, except for the high expression of *GhNPR1D* at the 20 DPA fiber stage. *GhNPR6A* and *GhNPR6D* were highly expressed throughout the 0 DPA to 25 DPA fiber stages, except for *GhNPR6A* at 0 DPA and *GhNPR6D* at 10 DPA. *GhNPR3A.2* and *GhNPR3D.2* were highly expressed throughout the 0DPA to 20DPA fiber stages, except for *GhNPR3A.2* in 10 DPA. The transcriptomic analysis of the fiber also suggests that *GhNPR5A* and *GhNPR5D* were highly expressed at the 5 DPA and 10 DPA fiber stages. We can broadly conclude from the in silico analysis of the transcriptomic data of the cotton fiber that a few cotton *NPR1*-like genes are probably involved in various stages of the fiber development.

### 2.6. Expression Analysis of the Cotton NPR1-Like Gene Family in Developmental Tissue

**Figure 8:**
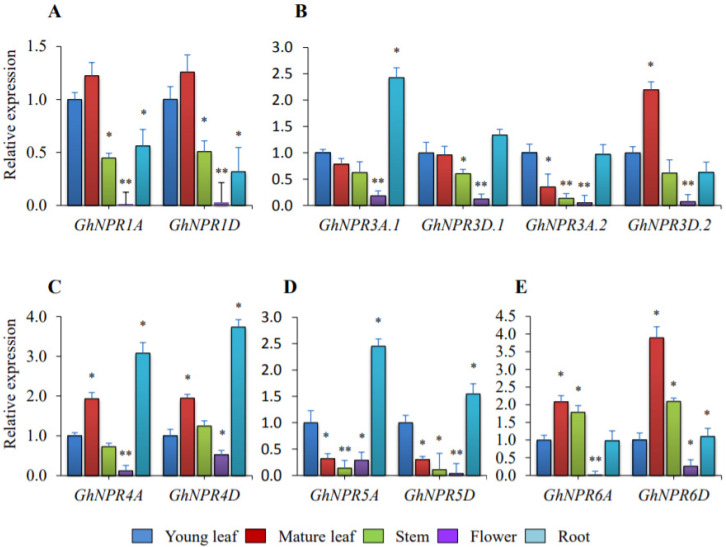
Real-time PCR for *GhNPR1*-like genes in different development tissues. The relative expression of *GhNPR1*-like genes in young leaf, mature leaf, stem, flower, and root of *G. hirsutum*. (**A**) Expression analysis of *GhNPR1*; (**B**) Expression analysis of *GhNPR3*; (**C**) Expression analysis of *GhNPR4*; (**D**) Expression analysis of *GhNPR5*; (**E**) Expression analysis of *GhNPR6*. Internal control gene *GhUBQ4* was used to normalize the expression of *GhNPR*-like genes. Data are the mean ± SE of three biological replicates with two technical replicates in each set. Significant differences are indicated by * *p* < 0.05; ** *p* < 0.005.

The gene expression analysis of representative *GhNPR* genes in different tissues—young leaf, mature leaf, stem, flower, and root—were determined by qRT-PCR analysis (Figure 8). The qRT-PCR analysis result showed that the gene expression level of all *GhNPR* genes was declined in flower, with respect to young leaf tissue. The expression level of *GhNPR1A* and *GhNPR1D* was low in stem, flower, and root tissue, in comparison with young leaf tissue. *GhNPR3A.2* showed relatively lower expression in the mature leaf and stem, whereas *GhNPR3A.1* and *GhNPR3D.2* exhibited higher expression in root and mature leaf tissue, respectively. The expression level of *GhNPR4A* and *GhNPR4D* was increased in the mature leaf and root as compared with the young leaf. The gene expression of *GhNPR5A* and *GhNPR5D* was downregulated in the mature leaf as compared with young leaf and upregulated in the root tissue, whereas *GhNPR6A* and *GhNPR6D* expression levels were increased in the mature leaf and stem as compared with the young leaf.

### 2.7. Expression Analysis of the Cotton NPR1-Like Gene Family in Different Fiber Stages

**Figure 9:**
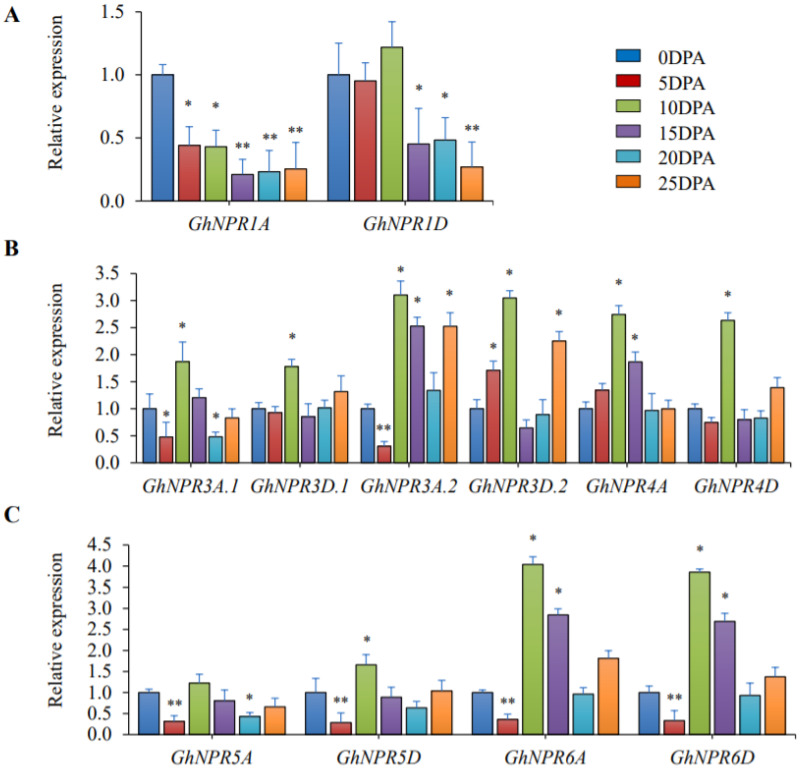
Real time PCR for *GhNPR1*-like genes in different fiber development stages. The relative expression of *GhNPR1*-like genes in different fiber development stages viz 0 days post-anthesis (DPA), 5 DPA, 10 DPA, 15 DPA, 20 DPA, and 25 DPA. (**A**) Expression analysis of *GhNPR1*; (**B**) Expression analysis of *GhNPR3* and *GhNPR4*; (**C**) Expression analysis of *GhNPR5* and *GhNPR6*. Internal control gene *GhUBQ4* was used to normalize the expression of *GhNPR1*-like genes. Data are the mean ± SE of three biological replicates with two technical replicates in each set. Significant differences are indicated by * *p* < 0.05; ** *p* < 0.005.

To address the impact of *GhNPR* genes on fiber development, qRT-PCR was performed at different fiber development stages: 0 DPA, 5 DPA, 10 DPA, 15 DPA, 20 DPA, and 25 DPA (Figure 9). The result of qRT-PCR analysis showed a decrease in expression of *GhNPR1A* in all fiber stages and *GhNPR1D* at later stages (15 DPA, 20 DPA, and 25 DPA) of fiber development (Figure 9A). Interestingly, enhanced expression of *GhNPR3*, *GhNPR4*, *GhNPR5D,* and *GhNPR6* was seen at 10 DPA, except for *GhNPR5A* (Figure 9B,C). Similarly, gene expression for *GhNPR3D.1*, *GhNPR3A.2/3D.2,* and *GhNPR6A* were increased at 25 DPA. However, at 15 DPA, expression of *GhNPR3A.2, GhNPR4A, GhNPR6A,* and *GhNPR6D* were increased, whereas the expression of *GhNPR3D.2* was decreased. Likewise, there is either a decrease or change in expression level at 5 DPA in *GhNPR* genes, except for *GhNPR3D.2,* which showed enhanced expression. It could be concluded that *GhNPR3*, *GhNPR4*, *GhNPR5,* and *GhNPR6* are involved in fiber development, contingent upon a detailed experimental validation.

### 2.8. Expression Analysis of the Cotton NPR1-Like Gene Family During SA-Induction

**Figure 10:**
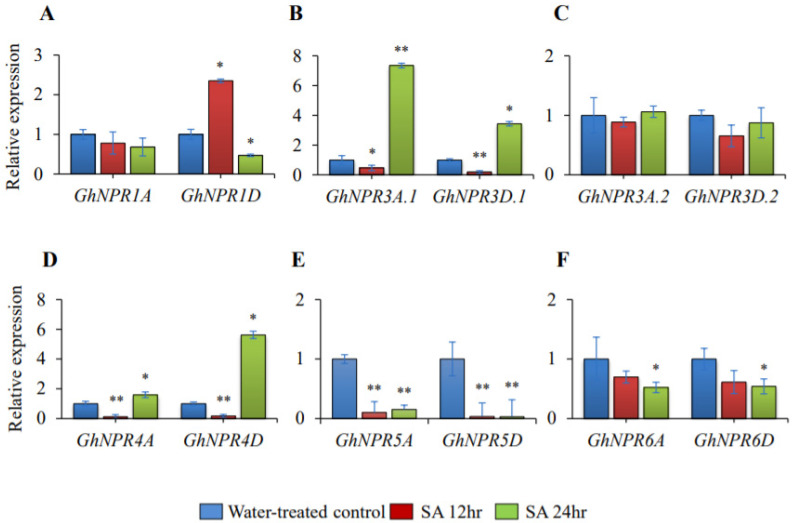
Salicylic acid effect on the *GhNPR1*-like gene expression pattern. The relative expression of *GhNPR* genes was estimated by qRT-PCR after 12 h and 24 h of SA induction in *G. hirsutum*. (**A**) Expression analysis of *GhNPR1*; (**B**) Expression analysis of *GhNPR3*; (**C**) Expression analysis of *GhNPR4*; (**D**) Expression analysis of *GhNPR5*; (**E**) Expression analysis of *GhNPR6*. Internal control gene *GhUBQ4* was used to normalize the expression in *G. hirsutum*. Data are the mean ± SE of three biological replicates with two technical replicates in each set. Significant differences are indicated by * *p* < 0.05.; ** *p* < 0.005.

The members of the *NPR1*-like gene family have a prominent role in the SAR pathway [38,39]. Therefore, the expression pattern of *G. hirsutum NPR1*-like genes were addressed in response to an exogenous application of salicylic acid (Figure 10, Figure S6). To assess the response from spraying 2 mM of salicylic acid on the leaves, expressions of the *GhNPR*-like genes were studied at 12 h and 24 h with respect to the water treated samples, which acted as a control. The qRT-PCR for samples with SA treatment showed a change in gene expression level for the *GhNPR1D* homolog, which was upregulated 2.7-fold (a statistically-significant change) at 12 h and downregulated 2.12-fold at 24 h (Figure 10A). The gene expression for *GhNPR3A.1*, *GhNPR3D.1*, *GhNPR4A,* and *GhNPR4D* showed downregulation at 12 h after SA induction, followed by an increase in the gene expression at 24 h of SA induction, significantly (Figure 10B–D). The gene expression of both the At and Dt subgenomes of *GhNPR5* and *GhNPR6* homologs was down-regulated at both 12 h and 24 h post-induction of salicylic acid, but the change for *GhNPR6A/D* gene expression was statistically insignificant at 12 h (Figure 10E–F). These results suggested that *GhNPR* genes *GhNPR1* to *GhNPR4* were regulated by SA, whereas *GhNPR5A/D* genes were repressed, indicating that SA has a different effect on different members of *G. hirsutum NPR1*-like genes.

## 3. Discussion

*NPR1*-like genes have been recognized as an important regulator of SA-dependent signaling and also of development pathways [5,11,40,41,42]. The present study is illustrative of the *NPR1*-like gene family in *Gossypium* species and provides a foundation for functional and evolutionary characterization of the cotton NPR1-like protein family. The protein and domain similarities between *Arabidopsis* and cotton were very high, suggestive of the evolutionary conservation of the proteins and their domain. Interestingly, the diploid genome tree of cotton *G. arboreum* (*GaNPR* genes) contains only five *NPR1*-like genes, whereas *G. raimondii* possesses six *NPR1*-like genes. Twelve *NPR1-*like genes in both allotetraploid cotton species of upland cotton (*GhNPR* genes) and Pima or sea-land cotton (*GbNPR* genes) were identified in an attempt to better understand signaling in the defense and fiber-related developmental response, which probably better facilitates these processes. Instead, *NPR1* and *BOP1* were identified earlier in *G. hirsutum* and their role in defense was identified, yet information about their isoforms and the functions of other members of the family is still elusive [23,39]. 

Perusal of the phylogenetic tree and the exon-intron structural organization suggested that the putative *NPR1*-like genes of all four cotton species, *Arabidopsis* and other known species (dicot, monocot, and lower group plants), were clustered in three different clades/groups (Figure 1, Figure 2 and Figure 3). As previously reported in *Arabidopsis*, members of clade I and II NPR proteins are implicated in activating or repressing the SAR signaling response, respectively, whereas clade III NPR members are mainly involved in leaf and flower development [41,43,44,45]. Identified NPR1 proteinds in *Gossypium* species have belonged to clade I, whereas NPR3 and NPR4 were clustered in clade II, and NPR5 and NPR6 belong to clade III. Those *Gossypium* species NPR proteins that fall in the same clade could function in a similar manner. Notably, NPR2 was not identified in either allotetraploid or diploid cotton, suggesting that it might be removed in the course of evolution. Further, phylogenetic analysis suggested that NPR1-like homologs probably originated from the bryophytes, as no evidence of these proteins exits in the algal genome, which is consistent with the previous findings [25]. We also identified several *NPR1*-like genes collinearity blocks among *G. hirsutum* (At and Dt- genome), *G. barbadense* (At and Dt- genome), *G. raimondii* (D-genome), and *G. arboreum* (A-genome) (Figure 4), revealing that segmental duplication occurred during expansion and evolution of *NPR1*-like genes in cotton. NPR1-like proteins comprise two essential domains mediating protein–protein interaction, the N-terminus BTB/POZ, and the central region ankyrin repeat domain, which are well conserved in all cotton species in this study. In contrast, the essential features characteristic of defense-related NPR1-like C-terminal domain were exclusively present in the group members of NPR1 to NPR4. Another DUF domain was also revealed in NPR-like proteins by CDD analysis, which is required to explore its function experimentally. 

GhNPR1A/D homologs possess a phosphorylation site at S11/S15 and a dephosphorylation site at S55/S59, which is similar to the distinguished features of AtNPR1/2 and orchestrates salicylic acid induction for the regulation of *PR1* gene expression and proteasome-mediated turnover of NPR1 (Figure 5) **[10,46]**. SA induction turns oligomeric NPR1 into monomeric NPR1, which leads to nuclear localization of the NPR1 protein [37,47]. In normal conditions, cysteine residues (marked as ● in Figure 5) at C82 (conserved in all cotton species) and C216 (present only in GhNPR1, GhNPR3.1, and GhNPR4) allow the NPR1 to exist in an oligomer form in the cytoplasm [36,48]. However, we also found some deviations from the conserved amino acid of *Arabidopsis* in cotton species, for example, cotton GhNPR1A/D proteins do not harbor the transactivation domain of *Arabidopsis* NPR1 at C521, but rather possess C529, which is essential for coactivator function and required for the binding of salicylic acid [7,9]. In addition, highly conserved arginine residues present in the penta-amino acid motif (LENRV), which are important for SA sensitivity in AtNPR1, AtNPR3, and AtNPR4, are also present in cotton NPR1, NPR3, and NPR4 [45]. Phosphorylated monomeric AtNPR1 at S589/T373 by SnRK2.8 which is significant for nuclear import, is well conserved in all cotton species and *Arabidopsis* NPR proteins [49]. The NLS motif is responsible for nuclear localization of AtNPR1/2, which is highly conserved in cotton NPR1A/1D, and a few basic amino acids (marked as **Δ** in Figure 5), and is also similar in the cotton NPR3/4 group of NPR proteins. However, the C-terminal of cotton NPR5/6 lacks certain indispensable properties of defense-related NPR1-like proteins, for instance, a clear bipartite NLS and the motif for NIMIN1/2 binding [37,50].

Distinct cis-elements present in the promoter or upstream regions of genes, which are binding sites for transcription factors, are also key factors in gene regulation [51,52,53]. The density of specific or diverse cis-elements present in promoter regions provide indications for the tissue-specific or stress-responsive expression patterns in diverse challenging environmental conditions [53,54,55,56]. Accumulating evidence has shown that the promoters of pathogen- or SA-inducible genes comprise a higher number of defense-related cis-elements [35,57,58]. By analogy, the promoters of development or tissue-specific genes comprise a higher number of cis-elements, accordingly [53,54]. In silico cis-element analysis suggested a higher enrichment of defense related cis-elements in the *GhNPR1* to *GhNPR4* group, whereas development-related motifs were enriched in the *GhNRP3* to *GhNPR6* group, suggesting that the putative role of the cotton NPR family is conserved throughout evolution in these important biological processes [11,40,41,42]. Chen et al., 2019, revealed that NPR1 protein positively regulates its expression by binding to its own promoter, and communicating with RNA polymerase II. AtNPR1 directly interacts and recruits cyclin-dependent kinase 8, which phosphorylates the RNA polymerase II C-terminal domain [59,60]. Similarly, a motif occurring in the *AtNPR1* promoter *GhNPR1A/D* contains several defense-regulated cis-elements, like W-box or Asf1 motifs for the binding of WRKY or TGA proteins, respectively, suggesting that *GhNPR1A/D* probably regulates its own expression.

In addition to the cotton seed, which is used for oil and protein, the natural cotton fiber is also a valuable resource as a raw material in industrial textile manufacturing, and is thereby a major contributor to a state’s economy. The in silico expression profiling of nine different tissues revealed that *GhNPR3A.1/D.1*, *GhNPR4A/D,* and *GhNPR6* have higher expression levels in the developmental tissue, relative to the *GhNPR1A/D*. Previously, several studies also reported the *NPR1*-like genes family expression pattern in various tissues at different levels [25,26,35,38,39,61,62,63]. The higher expression level of *GhNPR3A.2/D.2*, *GhNPR5A/D,* and *GhNPR6A/D* showed in fiber cell initiation and elongation stages (0–10 DPA), whereas *GhNPR3A.2/D.2* and *GhNPR6A/D* have higher expression levels during the rapid elongation stage (10–20 DPA). Interestingly, in silico expression profiling in the fiber reasonably correlated with qRT-PCR and analysis of the promoter cis-elements for *GhNPR3A.2/D.2, GhNPR5A/D,* and *GhNPR6A/D*, indicating that these genes are preferentially expressed in fiber and could potentially function for the initiation and elongation stages of fiber development. Additionally, for the proper development of the plant, its resistance/tolerance should be strong enough for a different kind of biotic or abiotic stress. *NPR* genes are mostly involved in the defense signaling pathway and their expression patterns are also variable at different time periods of SA induction or pathogen infestation [8,25,26,35,40]. The qRT-PCR analysis suggested that *GhNPR1D*, *GhNPR3A.1/D.1*, and *GhNPR4A/D* were significantly activated after SA exogenous treatment. Although Zhang et al., 2008, emphasized *GhNPR1* expression in SA induction at different time intervals, no information was provided about SA’s effect on *GhNPR1* homologs and their functions in fiber development [23]. Notably, *GhNPR3A.2/D.2*, *GhNPR5A/D,* and *GhNPR6A/D* were not expressed after exogenous SA treatment, which suggests no probable significant contribution in the defense pathway, and conversely showed participation in fiber development. Interestingly, a lower number of defense-regulated cis-elements were present in the upstream region of *GhNPR3A.2/D.2*, *GhNPR5A/D,* and *GhNPR6A/D* genes.

In conclusion, a set of homologs of cotton *NPR1*-like genes were identified in *G. hirsutum*, including six genes in the A-subgenome and six genes in the D-subgenome. Based on the gene and protein structural characteristics and comparison with homologs from other cotton and plant species, the NPR-like proteins were grouped into three different clades, signifying evolutionary conservation and functional divergence. Consequently, in silico and quantitative expression profile analysis of *GhNPR* genes suggest the probable functions of different aspects of *G. hirsutum* for biotic stress tolerance and various fiber development stages. Therefore, the present study could be a useful resource in using the cotton *NPR1*-like gene family for the future challenges, such as developing varieties for pathogen tolerance and higher fiber yield.

## 4. Materials and Methods

### 4.1. Plant Materials

*G. hirsutum* was grown in a soil mixture under long-day conditions (16/8 day/night cycle at 28 °C ± 2 °C, 1600 μmol m^−2^ sec^−1^) in a controlled glasshouse environment. *Arabidopsis thaliana* seeds (Col-0 ecotype) were stratified on Soilrite for four days at 4 °C, after which seeds were allowed to grow under controlled environmental conditions (22 °C ± 1 °C, 120 μmol m^−2^ sec^−1^, 16/8 day/night cycle). Leaves from *G. hirsutum* and *Arabidopsis* plants 8 weeks and 4 weeks post-sowing were taken for study, respectively.

### 4.2. Identification of NPR1-Like Genes Family in Cotton

The *Arabidopsis* Information Resource (TAIR) and Cotton Functional Genomics Database (CottonFGD) were used for the identification of the *NPR1*-like genes family in four *Gossypium* species—*G. hirsutum, G. barbadense, G. ramondii,* and *G. arboreum.* Protein sequences of all 6 *Arabidopsis* NPR family members AtNPR1 (At1G64280), AtNPR2 (At4G26120), AtNPR3 (At5G45110), AtNPR4 (At4G19660), AtNPR5 (At2G41370), and AtNPR6 (At3G57130) were utilized as queries to accomplish a protein–protein BLAST (BLASTP) program for the protein sequences of all 4 *Gossypium* species.

### 4.3. Protein Structure Analysis and Domain Distribution

Multiple sequence alignment was performed using identified putative cotton NPR and *Arabidopsis* NPR proteins with Clustal-X version 2 [64]. The locations of conserved BTB/POZ, ankyrin repeat domain, transactivation domain, and nuclear localization sequences (NLS) were determined using the Conserved Domain Database [37,65]. The predicted NPR1-like sequences were further confirmed for the presence of the conserved domain of NPR family members—BTB/POZ and ankyrin repeat domain through SMART and pfam databases. Percentages of amino acid identity and similarity between *Arabidopsis* and cotton sequences were identified by BioEdit software.

### 4.4. Physical Property Analysis of Cotton NPR Genes

The parameters of the identified putative cotton *NPR* genes—deduced amino acid length, molecular weight, theoretical pI, and grand average of hydropathy (GRAVY)—were obtained from the CottonFGD.

### 4.5. Analysis of Gene Structure and Chromosomal Localization 

The chromosomal localization of putative *G. hirsutum NPR* genes in A- and D-subgenomes to particular chromosomes, distinctly, was marked using Mapchart 2.3 software. For the gene structure analysis, coordinates of exon-intron for cotton and *Arabidopsis NPR1* gene families were diagrammatically represented by the Gene Structure Display Server 2.0 [66].

### 4.6. Phylogenetic and Synteny Analysis 

The protein sequences of NPR proteins from different plant species were retrieved and their multiple sequence alignment (MSA) were carried out with identified cotton NPR proteins using Clustal-X version 2 with default parameters. Subsequently, the obtained aligned protein sequences were used as inputs to construct unrooted phylogenetic trees following the neighbor-joining method with a Jones–Taylor–Thornton model and pairwise gap deletion by MEGA-X software with 1000 bootstrap replicates [67]. Other *NPR1*-like gene family homologs from different plant species were identified by executing BLASTP search or from published research articles [24,25,27,28] by taking protein sequences of 6 *Arabidopsis* NPR1 family members as a query for generation of a phylogenetic tree. MCScanX software was used for the syntenic analysis with the default parameters [68]. Circos was used to plot the segmental duplication events diagram on chromosomes [69].

### 4.7. Conserved Cis-Element Analysis in the Promoter

Sequences from the 2 kb upstream region of translation sites were retrieved for *GhNPR* genes from CottonFGD and AtNPR genes through the TAIR database for identification of the cis-regulatory elements. The promoter cis-regulatory elements present in the *NPR1*-like genes were identified by PLACE and PlantCare [70,71].

### 4.8. Analysis of RNA-Seq Data for Putative GhNPR Genes in Different Tissues and Fiber Development Stages

The RNA-Seq data of *G. hirsutum* TM1 in different tissues and fiber development stages were downloaded from NCBI (PRJNA248163). The obtained raw reads were quality filtered and further aligned to the reference genome of *G. hirsutum* using TopHat2 with default parameters [72]. The aligned reads’ fragments per kilobase million (FPKM) values were identified and heat maps were generated by Multi Experiment Viewer (MEV V.4.9.0) [73,74].

### 4.9. RNA Extraction and Real-Time PCR Analysis

To evaluate the putative *GhNPR* gene expression in the different fiber development stages, the flower was tagged on the day of flowering. The fiber tissues were harvested at 0 DPA (ovule), 5 DPA, 10 DPA, 15 DPA, 20 DPA, and 25 DPA. Similarly, samples were also collected for RNA isolation from different tissues: young leaf (top leaves from 8-week-old plants), mature leaf (lowest leaves from 8-week-old plants), stem (8-week-old plants), flower (10-week-old plants), and root (8-week-old plants) of *G. hirsutum.* For the induction of salicylic acid, 2 mM of salicylic acid was sprayed on *G. hirsutum* leaf tissue and harvested at 12 h and 24 h after induction for RNA extraction. Similarly, 2 mM of salicylic acid was sprayed and *Arabidopsis* leaf tissue was harvested after 12 h from 30 DAS (days after sowing) for RNA extraction [75]. RNA was isolated by the Spectrum Plant Total RNA Kit (Sigma-Aldrich). For the removal of DNA contamination, approx. 10 μg of isolated total RNA was treated with DNase1 (Turbo DNA-free, Ambion). The 2 µg of DNase1-treated RNA was used to make cDNA by SuperScript II RT-kit (Invitrogen, Life technologies). The quantitative reaction was executed on the 7500 Fast Real-Time PCR system (Applied Biosystem) by a fast SYBR Green master mix. Three biological and two technical replicates were taken for each experiment, and the 2^-∆∆CT^ method was used for analyzing the relative expression of target genes. For the validation of all genes, gene-specific primers (forward and reverse) were made by Primer Express 3.0.1 software (Applied Biosystems) (Table S1). Similarly, Real-time PCR analysis was also done for *AtNPR* genes in leaves of water- and salicylic acid-treated *Arabidopsis thaliana* by using the same procedure described above. Internal control genes like *GhUbiquitin4* [76] and *AtUbiquitin10* (At4g05320) [51] were used for normalizing the expression level of target genes in *GhNPR* and *AtNPR* genes, respectively.

## Figures and Tables

**Table 1:** Classification of nonexpressor of pathogenesis-related (NPR) homologs in cotton.

*Arabidopsis*		Tetraploid Cotton				Diploid Cotton			
		*G. hirsutum*		*G. barbadense*		*G. arboreum*		*G. raimondii*	
Gene Name	Gene ID	Gene Name	Gene ID	Gene Name	Gene ID	Gene Name	Gene ID	Gene Name	Gene ID
*NPR1*	AT1G64280	*GhNPR1A*	GH_A08G2791	*GbNPR1A*	GB_A08G2903	*GaNPR1*	Ga08G2883	*GrNPR1*	Gorai.004G284300
		*GhNPR1D*	GH_D08G2784	*GbNPR1D*	GB_D08G2893	*-*	-	*-*	-
*NPR2*	AT4G26120	*-*	-	*-*	-	*-*	-	*-*	-
*NPR3*	AT5G45110	*GhNPR3A.1*	GH_A09G0955	*GbNPR3A.1*	GB_A09G1067	*GaNPR3*	Ga09G0926	*GrNPR3.1*	Gorai.006G091900
		*GhNPR3D.1*	GH_D09G0911	*GbNPR3D.1*	GB_D09G0919	*-*	-	*GrNPR3.2*	Gorai.006G009000
		*GhNPR3A.2*	GH_A09G0085	*GbNPR3A.2*	GB_A09G0108	*-*	-	*-*	-
		*GhNPR3D.2*	GH_D09G0089	*GbNPR3D.2*	GB_D09G0086	*-*	-	*-*	-
*NPR4*	AT4G19660	*GhNPR4A*	GH_A10G0470	*GbNPR4A*	GB_A10G0469	*GaNPR4*	Ga10G2594	*GrNPR4*	Gorai.011G050200
		*GhNPR4D*	GH_D10G0496	*GbNPR4D*	GB_D10G0482	*-*	-	*-*	-
*NPR5*	AT2G41370	*GhNPR5A*	GH_A01G2100	*GbNPR5A*	GB_A01G2205	*GaNPR5*	Ga02G1425	*GrNPR5*	Gorai.002G226700
		*GhNPR5D*	GH_D01G2194	*GbNPR5D*	GB_D01G2289	*-*	-	*-*	-
*NPR6*	AT3G57130	*GhNPR6A*	GH_A09G1355	*GbNPR6A*	GB_A09G1470	*GaNPR6*	Ga09G1363	*GrNPR6*	Gorai.006G133600
		*GhNPR6D*	GH_D09G1306	*GbNPR6D*	GB_D09G1311	*-*	-	*-*	-

## Supplementary Materials

The following are available online at https://www.mdpi.com/2223-7747/9/8/999/s1, Table S1: List of primers, Table S2: Locus or Protein ID, Table S3: Protein similarity, Table S4: Protein and gene features, Table S5: Cis-regulatory elements in *Arabidopsis* and Cotton *NPR* genes, Figure S1: The exon-intron structure of *NPR1*-like genes in *G. raimondii, G. arboreum,* and *G. barbadense*, Figure S2: Genomic localization of *NPR1*-like genes in the allotetraploid *G. hirsutum,* Figure S3: Multiple sequence alignment of *G. arboreum* with *Arabidopsis* NPR proteins, Figure S4: Multiple sequence alignment of *G. raimondii* with *Arabidopsis* NPR proteins, Figure S5: Multiple sequence alignment of G. *barbadense* with *Arabidopsis* NPR proteins, Figure S6: Salicylic acid effect on the *NPR1*-like gene expression pattern in *Arabidopsis*

## References

[1] Durrant W.E., Dong X. (2004). Systemic acquired resistance. Annu. Rev. Phytopathol..

[2] Fu Z.Q., Dong X. (2013). Systemic acquired resistance: Turning local infection into global defense. Annu. Rev. Plant Biol..

[3] An C., Mou Z. (2011). Salicylic acid and its function in plant immunity. J. Integr. Plant Biol..

[4] Malamy J., Carr J.P., Klessig D.F., Raskin I. (1990). Salicylic Acid: A likely endogenous signal in the resistance response of tobacco to viral infection. Science.

[5] Cao H., Bowling S.A., Gordon A.S., Dong X. (1994). Characterization of an Arabidopsis Mutant That Is Nonresponsive to Inducers of Systemic Acquired Resistance. Plant Cell.

[6] Delaney T.P., Friedrich L., Ryals J.A. (1995). Arabidopsis signal transduction mutant defective in chemically and biologically induced disease resistance. Proc. Natl. Acad. Sci. USA.

[7] Wu Y., Zhang D., Chu J.Y., Boyle P., Wang Y., Brindle I.D., De Luca V., Després C. (2012). The Arabidopsis NPR1 Protein Is a Receptor for the Plant Defense Hormone Salicylic Acid. Cell Rep..

[8] Zhang X., Chen S., Mou Z. (2010). Nuclear localization of NPR1 is required for regulation of salicylate tolerance, isochorismate synthase 1 expression and salicylate accumulation in Arabidopsis. J. Plant Physiol..

[9] Rochon A., Boyle P., Wignes T., Fobert P.R., Despres C. (2006). The coactivator function of Arabidopsis NPR1 requires the core of its BTB/POZ domain and the oxidation of C-terminal cysteines. Plant Cell.

[10] Spoel S.H., Mou Z., Tada Y., Spivey N.W., Genschik P., Dong X. (2009). Proteasome-mediated turnover of the transcription coactivator NPR1 plays dual roles in regulating plant immunity. Cell.

[11] Hepworth S.R., Zhang Y., McKim S., Li X., Haughn G.W. (2005). BLADE-ON-PETIOLE-dependent signaling controls leaf and floral patterning in Arabidopsis. Plant Cell.

[12] Makandar R., Essig J.S., Schapaugh M.A., Trick H.N., Shah J. (2006). Genetically engineered resistance to Fusarium head blight in wheat by expression of Arabidopsis NPR1. Mol. Plant Microbe Interact..

[13] Dutt M., Barthe G., Irey M., Grosser J. (2015). Transgenic Citrus Expressing an Arabidopsis NPR1 Gene Exhibit Enhanced Resistance against Huanglongbing (HLB.; Citrus Greening). PLoS ONE.

[14] Zhang X.D., Francis M.I., Dawson W.O., Graham J.H., Orbovic V., Triplett E.W., Mou Z.L. (2010). Over-expression of the Arabidopsis NPR1 gene in citrus increases resistance to citrus canker. Eur. J. Plant Pathol..

[15] Chern M.S., Fitzgerald H.A., Yadav R.C., Canlas P.E., Dong X., Ronald P.C. (2001). Evidence for a disease-resistance pathway in rice similar to the NPR1-mediated signaling pathway in Arabidopsis. Plant J..

[16] Molla K.A., Karmakar S., Chanda P.K., Sarkar S.N., Datta S.K., Datta K. (2016). Tissue-specific expression of Arabidopsis NPR1 gene in rice for sheath blight resistance without compromising phenotypic cost. Plant Sci..

[17] Lin W.C., Lu C.F., Wu J.W., Cheng M.L., Lin Y.M., Yang N.S., Black L., Green S.K., Wang J.F., Cheng C.P. (2004). Transgenic tomato plants expressing the Arabidopsis NPR1 gene display enhanced resistance to a spectrum of fungal and bacterial diseases. Transgenic Res..

[18] Potlakayala S.D., DeLong C., Sharpe A., Fobert P.R. (2007). Conservation of NON-EXPRESSOR OF PATHOGENESIS-RELATED GENES1 function between Arabidopsis thaliana and Brassica napus. Physiol. Mol. Plant Pathol..

[19] Meur G., Budatha M., Srinivasan T., Rajesh Kumar K.R., Dutta Gupta A., Kirti P.B. (2008). Constitutive expression of Arabidopsis NPR1 confers enhanced resistance to the early instars of Spodoptera litura in transgenic tobacco. Physiol. Plant.

[20] Wally O., Jayaraj J., Punja Z.K. (2009). Broad-spectrum disease resistance to necrotrophic and biotrophic pathogens in transgenic carrots (Daucus carota L.) expressing an Arabidopsis NPR1 gene. Planta.

[21] Kumar V., Joshi S.G., Bell A.A., Rathore K.S. (2013). Enhanced resistance against Thielaviopsis basicola in transgenic cotton plants expressing Arabidopsis NPR1 gene. Transgenic Res..

[22] Parkhi V., Kumar V., Campbell L.M., Bell A.A., Shah J., Rathore K.S. (2010). Resistance against various fungal pathogens and reniform nematode in transgenic cotton plants expressing Arabidopsis NPR1. Transgenic Res..

[23] Zhang Y., Wang X., Cheng C., Gao Q., Liu J., Guo X. (2008). Molecular cloning and characterization of GhNPR1, a gene implicated in pathogen responses from cotton (Gossypium hirsutum L.). Biosci. Rep..

[24] Backer R., Mahomed W., Reeksting B.J., Engelbrecht J., Ibarra-Laclette E., van den Berg N. (2015). Phylogenetic and expression analysis of the NPR1-like gene family from Persea americana (Mill.). Front. Plant Sci..

[25] Peraza-Echeverria S., Santamaría J.M., Fuentes G., de los Ángeles Menéndez-Cerón M., Vallejo-Reyna M.Á., Herrera-Valencia V.A. (2012). The NPR1 family of transcription cofactors in papaya: Insights into its structure, phylogeny and expression. Genes Genom..

[26] Liu X., Liu Z., Niu X., Xu Q., Yang L. (2019). Genome-Wide Identification and Analysis of the NPR1-Like Gene Family in Bread Wheat and Its Relatives. Int. J. Mol. Sci..

[27] Zhang J., Jiao P., Zhang C., Tong X., Wei Q., Xu L. (2016). Apple NPR1 homologs and their alternative splicing forms may contribute to SA and disease responses. Tree Genet. Genomes.

[28] Shu L.J., Liao J.Y., Lin N.C., Chung C.L. (2018). Identification of a strawberry NPR-like gene involved in negative regulation of the salicylic acid-mediated defense pathway. PLoS ONE.

[29] Phillips A.Z., Berry J.C., Wilson M.C., Vijayaraghavan A., Burke J., Bunn J.I., Allen T.W., Wheeler T., Bart R.S. (2017). Genomics-enabled analysis of the emergent disease cotton bacterial blight. PLoS Genet..

[30] Dixit G., Srivastava A., Rai K.M., Dubey R.S., Srivastava R., Verma P.C. (2020). Distinct defensive activity of phenolics and phenylpropanoid pathway genes in different cotton varieties toward chewing pests. Plant Signal. Behav..

[31] Hu Y., Chen J., Fang L., Zhang Z., Ma W., Niu Y., Ju L., Deng J., Zhao T., Lian J. (2019). Gossypium barbadense and Gossypium hirsutum genomes provide insights into the origin and evolution of allotetraploid cotton. Nat. Genet..

[32] Li F., Fan G., Wang K., Sun F., Yuan Y., Song G., Li Q., Ma Z., Lu C., Zou C. (2014). Genome sequence of the cultivated cotton Gossypium arboreum. Nat. Genet..

[33] Wang K., Wang Z., Li F., Ye W., Wang J., Song G., Yue Z., Cong L., Shang H., Zhu S. (2012). The draft genome of a diploid cotton Gossypium raimondii. Nat. Genet..

[34] Zhang T., Hu Y., Jiang W., Fang L., Guan X., Chen J., Zhang J., Saski C.A., Scheffler B.E., Stelly D.M. (2015). Sequencing of allotetraploid cotton (Gossypium hirsutum L. acc. TM-1) provides a resource for fiber improvement. Nat. Biotechnol..

[35] Backer R., Naidoo S., van den Berg N. (2019). The NONEXPRESSOR OF PATHOGENESIS-RELATED GENES 1 (NPR1) and Related Family: Mechanistic Insights in Plant Disease Resistance. Front. Plant Sci..

[36] Mou Z., Fan W., Dong X. (2003). Inducers of plant systemic acquired resistance regulate NPR1 function through redox changes. Cell.

[37] Kinkema M., Fan W., Dong X. (2000). Nuclear localization of NPR1 is required for activation of PR gene expression. Plant Cell.

[38] Castelló M.J., Medina-Puche L., Lamilla J., Tornero P. (2018). NPR1 paralogs of Arabidopsis and their role in salicylic acid perception. PLoS ONE.

[39] Zhang Z., Wang P., Luo X., Yang C., Tang Y., Wang Z., Hu G., Ge X., Xia G., Wu J. (2019). Cotton plant defence against a fungal pathogen is enhanced by expanding BLADE-ON-PETIOLE1 expression beyond lateral-organ boundaries. Commun. Biol..

[40] Pajerowska-Mukhtar K.M., Emerine D.K., Mukhtar M.S. (2013). Tell me more: Roles of NPRs in plant immunity. Trends Plant Sci..

[41] Ha C.M., Jun J.H., Nam H.G., Fletcher J.C. (2004). BLADE-ON-PETIOLE1 encodes a BTB/POZ domain protein required for leaf morphogenesis in Arabidopsis thaliana. Plant Cell Physiol..

[42] Zhang Y., Cheng Y.T., Qu N., Zhao Q., Bi D., Li X. (2006). Negative regulation of defense responses in Arabidopsis by two NPR1 paralogs. Plant J..

[43] Wang Y., Salasini B.C., Khan M., Devi B., Bush M., Subramaniam R., Hepworth S.R. (2019). Clade I TGACG-Motif Binding Basic Leucine Zipper Transcription Factors Mediate BLADE-ON-PETIOLE-Dependent Regulation of Development. Plant Physiol..

[44] Innes R. (2018). The Positives and Negatives of NPR: A Unifying Model for Salicylic Acid Signaling in Plants. Cell.

[45] Ding Y., Sun T., Ao K., Peng Y., Zhang Y., Li X., Zhang Y. (2018). Opposite Roles of Salicylic Acid Receptors NPR1 and NPR3/NPR4 in Transcriptional Regulation of Plant Immunity. Cell.

[46] Saleh A., Withers J., Mohan R., Marqués J., Gu Y., Yan S., Zavaliev R., Nomoto M., Tada Y., Dong X. (2015). Posttranslational Modifications of the Master Transcriptional Regulator NPR1 Enable Dynamic but Tight Control of Plant Immune Responses. Cell Host Microbe.

[47] Withers J., Dong X. (2016). Posttranslational Modifications of NPR1: A Single Protein Playing Multiple Roles in Plant Immunity and Physiology. PLoS Pathog..

[48] Tada Y., Spoel S.H., Pajerowska-Mukhtar K., Mou Z., Song J., Wang C., Zuo J., Dong X. (2008). Plant immunity requires conformational changes [corrected] of NPR1 via S-nitrosylation and thioredoxins. Science.

[49] Lee H.J., Park Y.J., Seo P.J., Kim J.H., Sim H.J., Kim S.G., Park C.M. (2015). Systemic Immunity Requires SnRK2.8-Mediated Nuclear Import of NPR1 in Arabidopsis. Plant Cell.

[50] Maier F., Zwicker S., Hückelhoven A., Meissner M., Funk J., Pfitzner A.J.P., Pfitzner U.M. (2011). NONEXPRESSOR OF PATHOGENESIS-RELATED PROTEINS1 (NPR1) and some NPR1-related proteins are sensitive to salicylic acid. Mol. Plant Pathol..

[51] Srivastava R., Rai K.M., Pandey B., Singh S.P., Sawant S.V. (2015). Spt-Ada-Gcn5-Acetyltransferase (SAGA) Complex in Plants: Genome Wide Identification, Evolutionary Conservation and Functional Determination. PLoS ONE.

[52] Srivastava R., Rai K.M., Srivastava M., Kumar V., Pandey B., Singh S.P., Bag S.K., Singh B.D., Tuli R., Sawant S.V. (2014). Distinct Role of Core Promoter Architecture in Regulation of Light Mediated Responses in Plant Genes. Mol. Plant.

[53] Biłas R., Szafran K., Hnatuszko-Konka K., Kononowicz A.K. (2016). Cis-regulatory elements used to control gene expression in plants. Plant Cell Tissue Organ Cul. (PCTOC).

[54] Pandey B., Prakash P., Verma P.C., Srivastava R. (2019). Regulated gene expression by synthetic modulation of the promoter architecture in plants. Current Developments in Biotechnology and Bioengineering: Synthetic Biology, Cell Engineering and Bioprocessing Technologies.

[55] Srivastava R., Srivastava R., Singh U.M. (2014). Understanding the patterns of gene expression during climate change. Climate Change Effect on Crop Productivity.

[56] Liu W., Stewart C.N. (2016). Plant synthetic promoters and transcription factors. Curr. Opin. Biotechnol..

[57] Srivastava R., Rai K.M., Srivastava R. (2018). Plant Biosynthetic Engineering Through Transcription Regulation: An Insight into Molecular Mechanisms During Environmental Stress. Biosynthetic Technology and Environmental Challenges.

[58] Rushton P.J., Somssich I.E. (1998). Transcriptional control of plant genes responsive to pathogens. Curr. Opin. Plant Biol..

[59] Chen J., Mohan R., Zhang Y., Li M., Chen H., Palmer I.A., Chang M., Qi G., Spoel S.H., Mengiste T. (2019). NPR1 Promotes Its Own and Target Gene Expression in Plant Defense by Recruiting CDK8. Plant Physiol..

[60] Srivastava R., Ahn S.H. (2015). Modifications of RNA polymerase II CTD: Connections to the histone code and cellular function. Biotechnol. Adv..

[61] Zhong X., Xi L., Lian Q., Luo X., Wu Z., Seng S., Yuan X., Yi M. (2015). The NPR1 homolog GhNPR1 plays an important role in the defense response of Gladiolus hybridus. Plant Cell Rep..

[62] Shi Z., Maximova S.N., Liu Y., Verica J., Guiltinan M.J. (2010). Functional analysis of the Theobroma cacao NPR1 gene in arabidopsis. BMC Plant Biol..

[63] Sandhu D., Tasma I.M., Frasch R., Bhattacharyya M.K. (2009). Systemic acquired resistance in soybean is regulated by two proteins, Orthologous to Arabidopsis NPR1. BMC Plant Biol..

[64] Larkin M.A., Blackshields G., Brown N.P., Chenna R., McGettigan P.A., McWilliam H., Valentin F., Wallace I.M., Wilm A., Lopez R. (2007). Clustal W and clustal X version 2.0. Bioinformatics.

[65] Marchler-Bauer A., Bo Y., Han L., He J., Lanczycki C.J., Lu S., Chitsaz F., Derbyshire M.K., Geer R.C., Gonzales N.R. (2017). CDD/SPARCLE: Functional classification of proteins via subfamily domain architectures. Nucleic Acids Res..

[66] Hu B., Jin J., Guo A.Y., Zhang H., Luo J., Gao G. (2015). GSDS 2.0: An upgraded gene feature visualization server. Bioinformatics.

[67] Kumar S., Stecher G., Li M., Knyaz C., Tamura K. (2018). MEGA X: Molecular Evolutionary Genetics Analysis across Computing Platforms. Mol. Biol. Evol..

[68] Wang Y., Tang H., Debarry J.D., Tan X., Li J., Wang X., Lee T.H., Jin H., Marler B., Guo H. (2012). MCScanX: A toolkit for detection and evolutionary analysis of gene synteny and collinearity. Nucleic Acids Res..

[69] Krzywinski M., Schein J., Birol I., Connors J., Gascoyne R., Horsman D., Jones S.J., Marra M.A. (2009). Circos: An information aesthetic for comparative genomics. Genome Res..

[70] Higo K., Ugawa Y., Iwamoto M., Korenaga T. (1999). Plant cis-acting regulatory DNA elements (PLACE) database: 1999. Nucleic Acids Res..

[71] Lescot M., Dehais P., Thijs G., Marchal K., Moreau Y., Van de Peer Y., Rouze P., Rombauts S. (2002). PlantCARE, a database of plant cis-acting regulatory elements and a portal to tools for in silico analysis of promoter sequences. Nucleic Acids Res..

[72] Kim D., Pertea G., Trapnell C., Pimentel H., Kelley R., Salzberg S.L. (2013). TopHat2: Accurate alignment of transcriptomes in the presence of insertions, deletions and gene fusions. Genome Biol..

[73] Howe E., Holton K., Nair S., Schlauch D., Sinha R., Quackenbush J. (2010). Mev: Multiexperiment viewer. Biomedical Informatics for Cancer Research.

[74] Trapnell C., Roberts A., Goff L., Pertea G., Kim D., Kelley D.R., Pimentel H., Salzberg S.L., Rinn J.L., Pachter L. (2012). Differential gene and transcript expression analysis of RNA-seq experiments with TopHat and Cufflinks. Nat. Protoc..

[75] Lodhi N., Ranjan A., Singh M., Srivastava R., Singh S.P., Chaturvedi C.P., Ansari S.A., Sawant S.V., Tuli R. (2008). Interactions between upstream and core promoter sequences determine gene expression and nucleosome positioning in tobacco PR-1a promoter. Biochim. Biophys. Acta.

[76] Artico S., Nardeli S.M., Brilhante O., Grossi-de-Sa M.F., Alves-Ferreira M. (2010). Identification and evaluation of new reference genes in Gossypium hirsutum for accurate normalization of real-time quantitative RT-PCR data. BMC Plant Biol..

