# Supplementary material for: Unravelling Cotton Nonexpressor of Pathogenesis-Related 1(NPR1)-Like Genes Family: Evolutionary Analysis and Putative Role in Fiber Development and Defense Pathway
Source: Plants (Basel). 2020 Aug 6;9(8):999. doi: 10.3390/plants9080999 (PMC7463611; doi:10.3390/plants9080999)
Supplement: Supplementary file 1 [file plants-09-00999-s001.zip › SupplementaryFigures mdpi-plants S1-S6.pptx]

## Slide 1
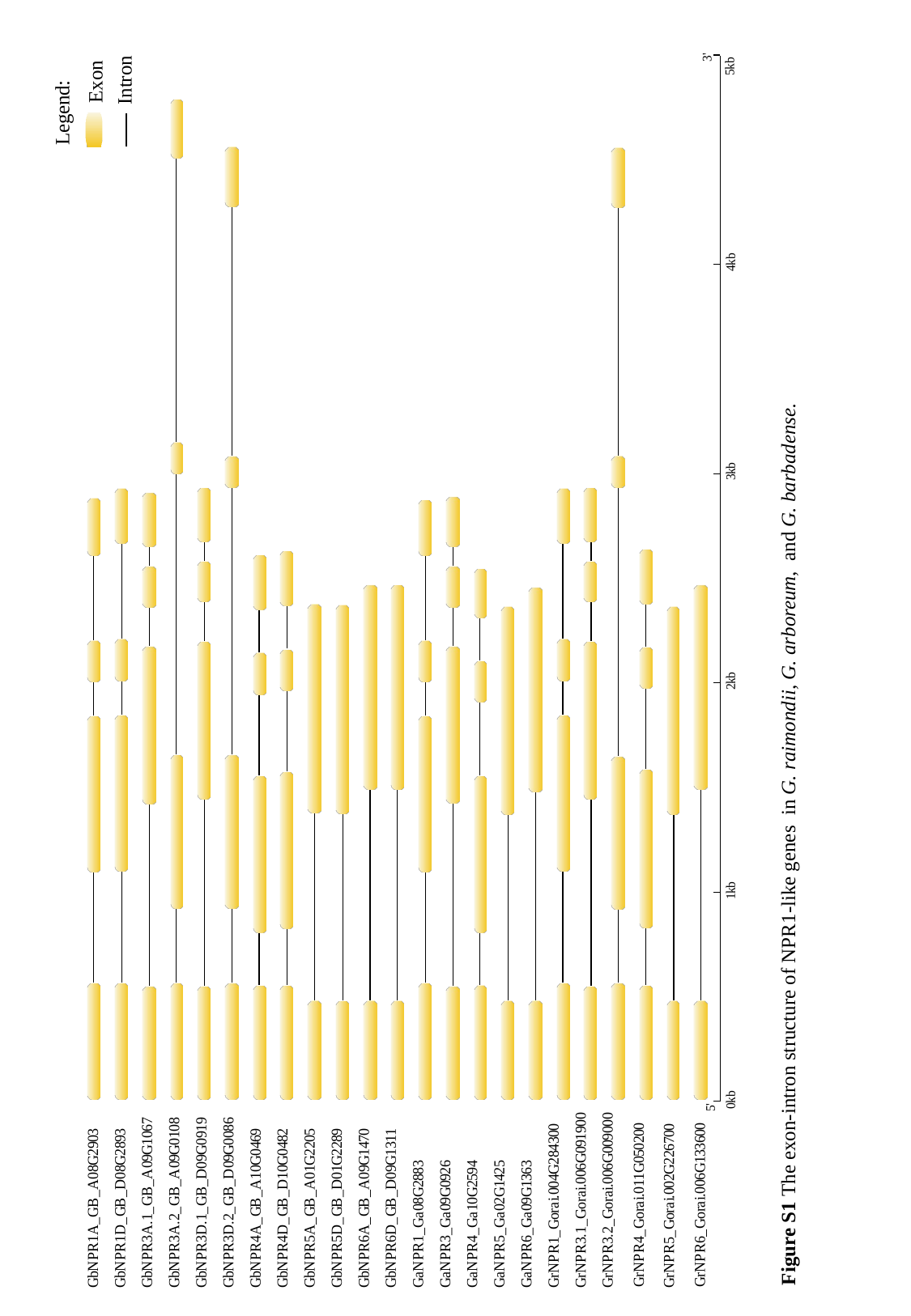

Legend:
Exon
Intron
Figure S1 The exon-intron structure of NPR1-like genes in G. raimondii, G. arboreum, and G. barbadense.

## Slide 2
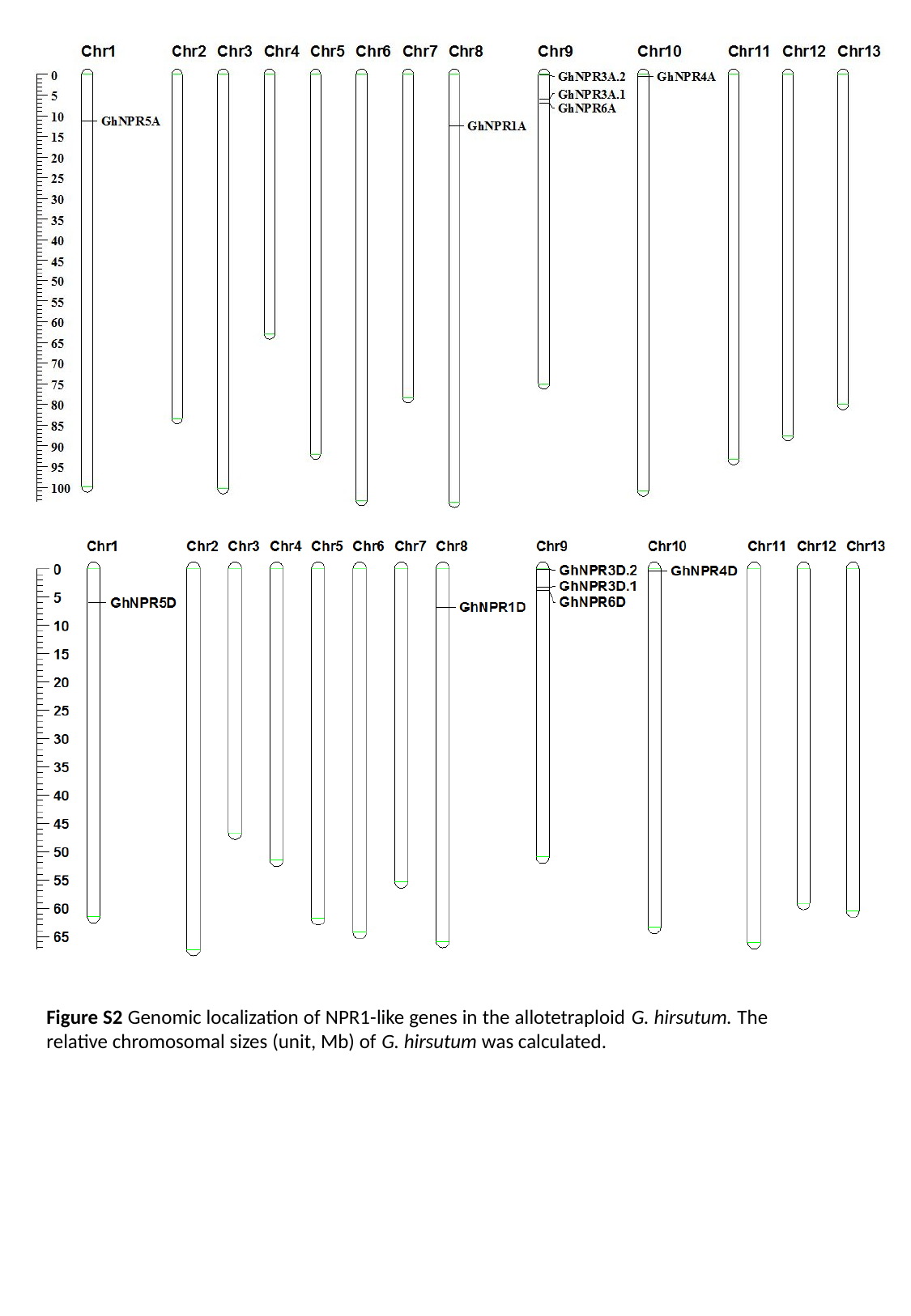

Figure S2 Genomic localization of NPR1-like genes in the allotetraploid G. hirsutum. The relative chromosomal sizes (unit, Mb) of G. hirsutum was calculated.

## Slide 3
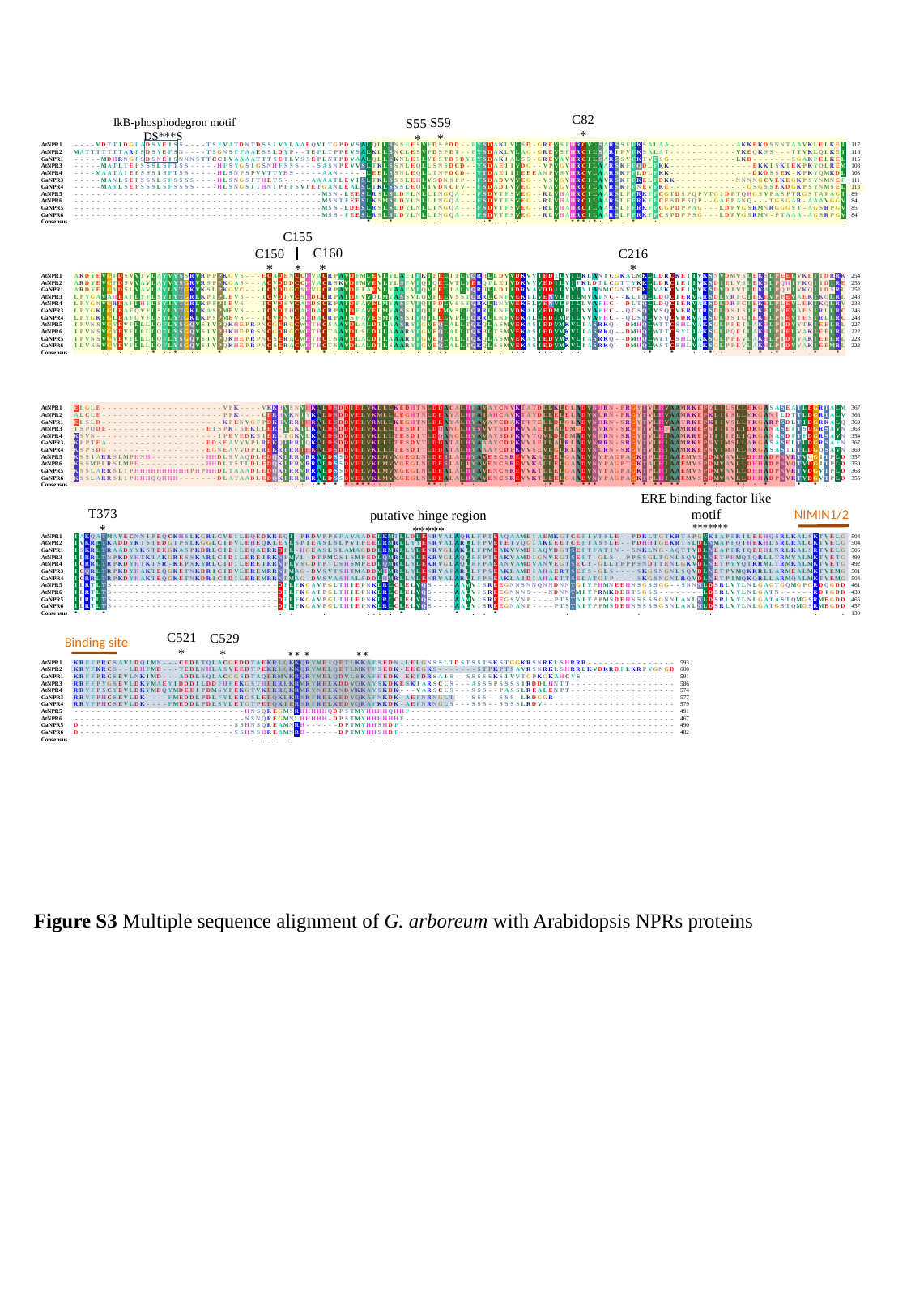

C82
*
S59
*
S55
 *
 IkB-phosphodegron motif
 DS***S
 C155
 *
 C160
 *
C150
*
C216
*
ERE binding factor like motif
 *******
T373
*
NIMIN1/2
putative hinge region
*****
C521
*
C529
*
 ** * **
Binding site
Figure S3 Multiple sequence alignment of G. arboreum with Arabidopsis NPRs proteins

## Slide 4
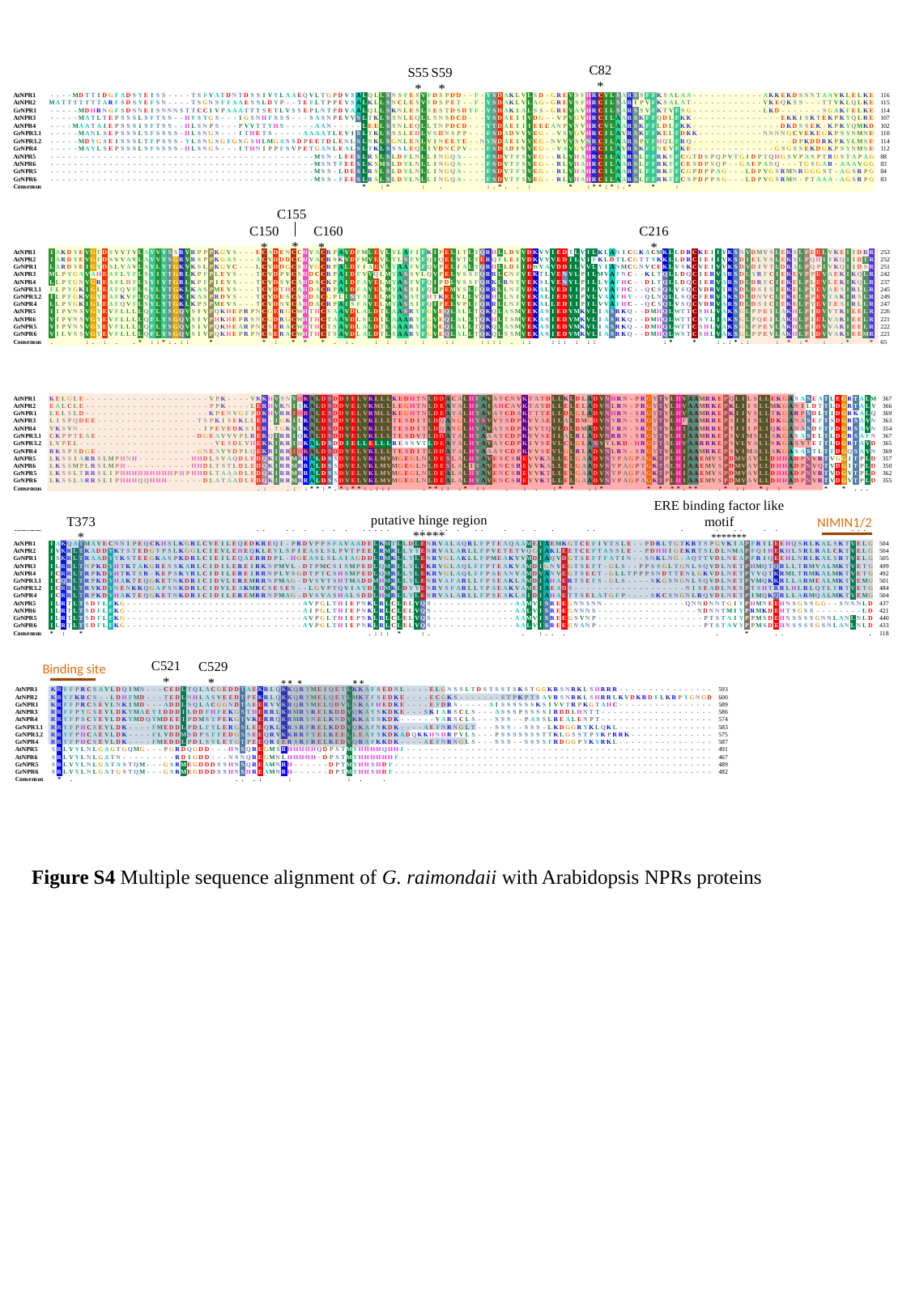

C82
*
S55
*
S59
*
C155
 *
C150
*
 C160
 *
C216
*
ERE binding factor like motif
 *******
putative hinge region
*****
T373
*
NIMIN1/2
C521
*
C529
*
 ** * **
Binding site
Figure S4 Multiple sequence alignment of G. raimondaii with Arabidopsis NPRs proteins

## Slide 5
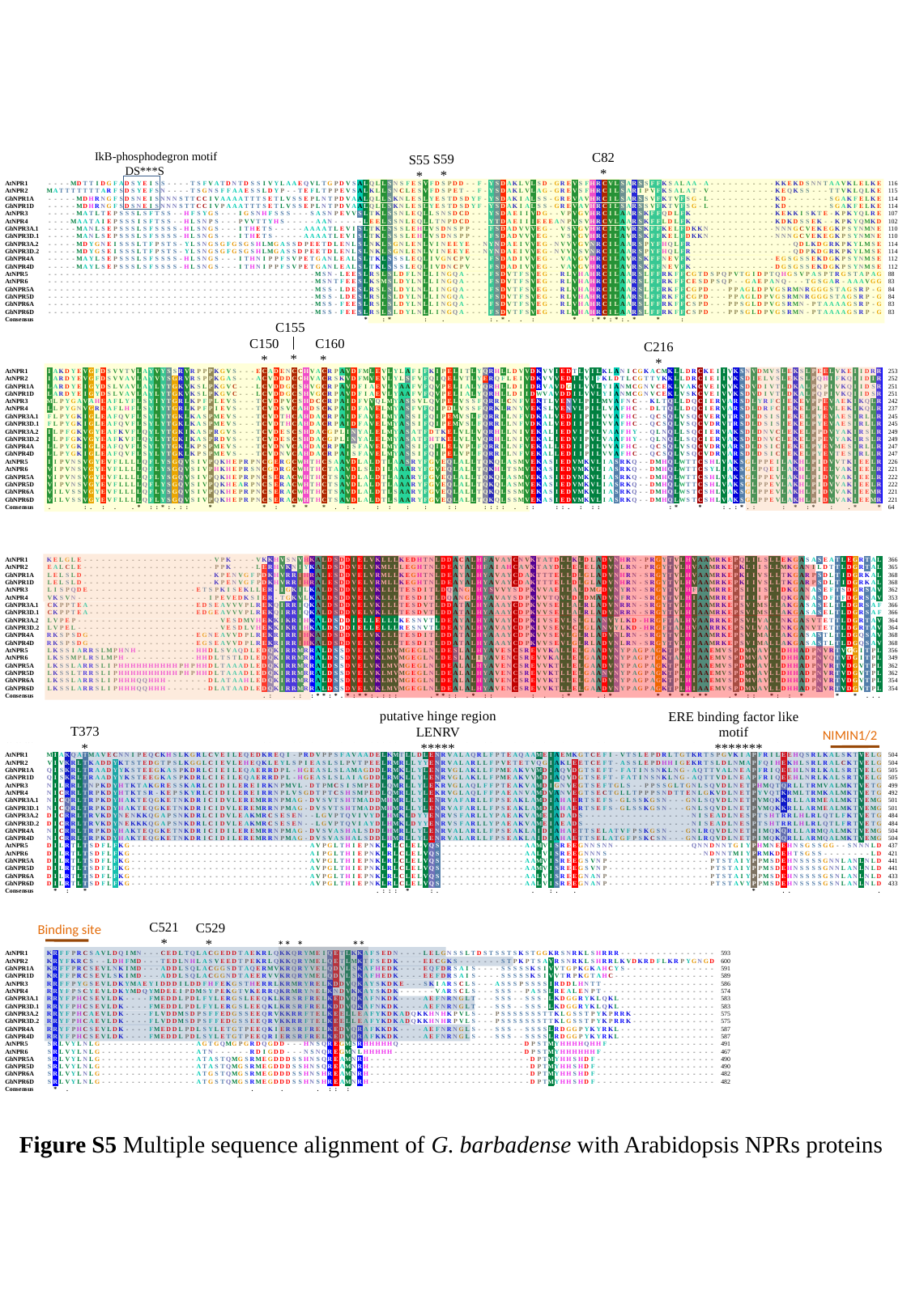

C82
*
 IkB-phosphodegron motif
 DS***S
S59
*
S55
*
C155
 *
C150
*
 C160
 *
C216
*
putative hinge region
LENRV
*****
ERE binding factor like motif
 *******
T373
*
NIMIN1/2
C521
*
C529
*
 ** * **
Binding site
Figure S5 Multiple sequence alignment of G. barbadense with Arabidopsis NPRs proteins

## Slide 6
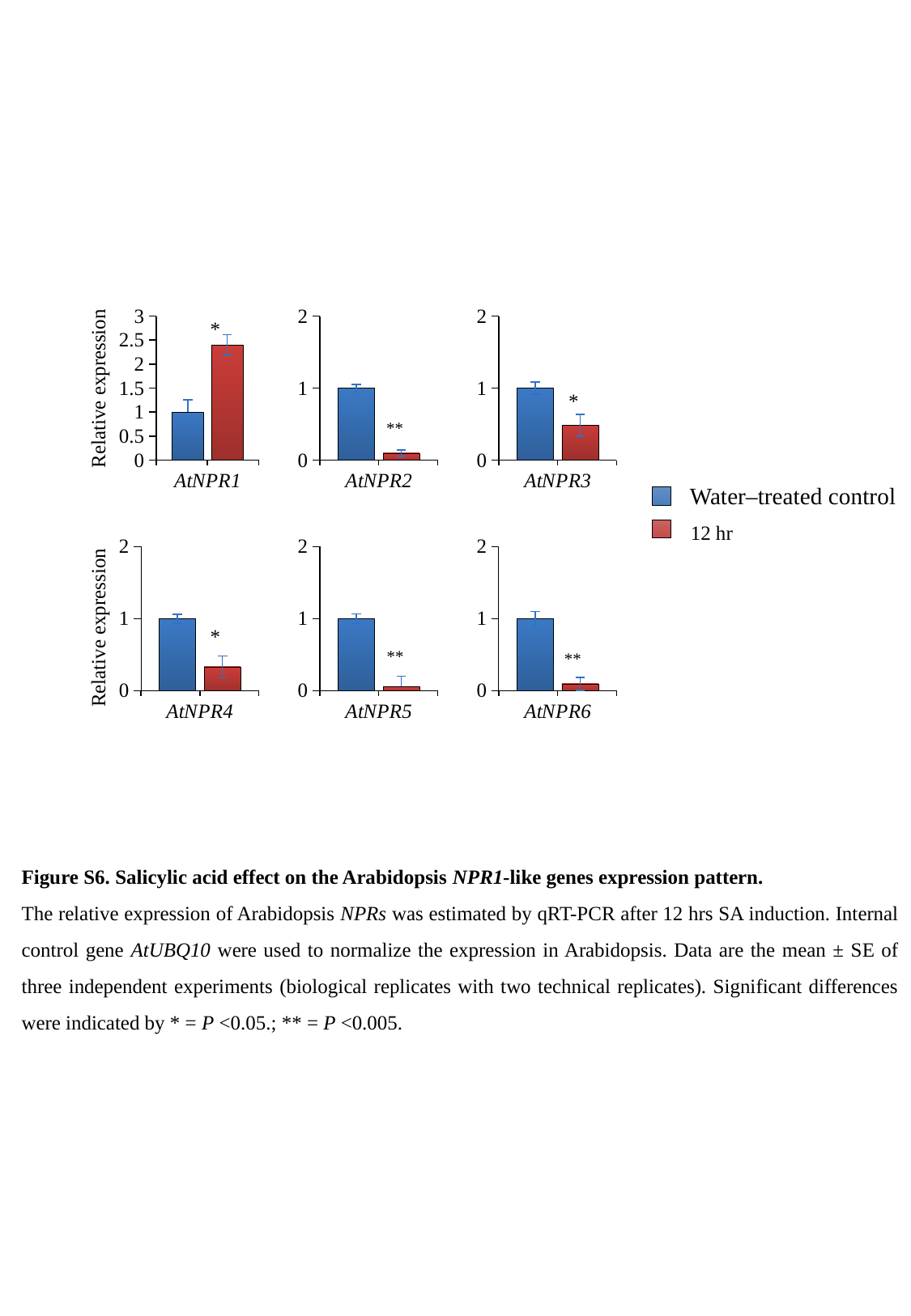

### Chart
| Category | 0 hr | 12 hr |
|---|---|---|
| AtNPR1 | 1.0 | 2.3985135535131836 |*
### Chart
| Category | 0 hr | 12 hr |
|---|---|---|
| AtNPR2 | 1.0 | 0.09714063361128047 |**
### Chart
| Category | 0 hr | 12 hr |
|---|---|---|
| AtNPR3 | 1.0 | 0.485435340757199 |*
Water–treated control
12 hr
### Chart
| Category | 0 hr | 12 hr |
|---|---|---|
| AtNPR4 | 1.0 | 0.330434287936662 |*
### Chart
| Category | 0 hr | 12 hr |
|---|---|---|
| AtNPR5 | 1.0 | 0.056059330404098766 |**
### Chart
| Category | 0 hr | 12 hr |
|---|---|---|
| AtNPR6 | 1.0 | 0.0935789744718636 |**
Relative expression
Relative expression
Figure S6. Salicylic acid effect on the Arabidopsis NPR1-like genes expression pattern.
The relative expression of Arabidopsis NPRs was estimated by qRT-PCR after 12 hrs SA induction. Internal control gene AtUBQ10 were used to normalize the expression in Arabidopsis. Data are the mean ± SE of three independent experiments (biological replicates with two technical replicates). Significant differences were indicated by * = P <0.05.; ** = P <0.005.
